# Significant association between vitamin D deficiency and sepsis: a systematic review and meta-analysis

**DOI:** 10.1186/s12871-015-0063-3

**Published:** 2015-06-04

**Authors:** Sikarin Upala, Anawin Sanguankeo, Nitipong Permpalung

**Affiliations:** 1 Department of Internal Medicine, Bassett Medical Center and Columbia University College of Physicians and Surgeons, Cooperstown, NY USA; 2 Department of Preventive and Social Medicine, Faculty of Medicine Siriraj Hospital, Mahidol University, Bangkok, Thailand

**Keywords:** Vitamin D deficiency, Sepsis, Systematic review, Meta-analysis

## Abstract

**Background:**

A number of observational studies have found an association between low vitamin D levels and risk of sepsis. We conducted a systematic review and meta-analysis to determine the overall estimate of risk.

**Methods:**

This was a systematic review and meta-analysis conducted by online searches (CENTRAL, PubMed/MEDLINE, and EMBASE) was registered in PROSPERO (CRD42014014767). Primary outcome was incidence, prevalence, relative risk or odds ratio of having sepsis or bloodstream infection between patients with vitamin D deficiency and controls.

**Results:**

The initial search yielded 647 articles. Twenty-one articles underwent full-length review and data were extracted from 10 observational studies. Pooled odds ratio of sepsis in participants with vitamin D deficiency was 1.78 (95 % confidence interval [CI] = 1.55 to 2.03, p < 0.01) compared with controls in studies that reported participant numbers and was 1.45 (95 % CI = 1.26 to 1.66, p < 0.01) in studies that reported an adjusted odds ratio of vitamin D deficiency for developing sepsis. Statistical between-study heterogeneity was low (I^2^ = 0 % and 5 %, respectively). Standardized mean difference of 25-hydroxyvitamin D levels in patients with sepsis and controls was −0.24 (95 % CI = −0.49 to 0.00, p = 0.05) and lower in the sepsis group compared with non-sepsis or control participants. The statistical between-study heterogeneity (I^2^) was 0 %.

**Conclusion:**

Vitamin D deficiency were associated with an increased susceptibility of sepsis.

**Electronic supplementary material:**

The online version of this article (doi:10.1186/s12871-015-0063-3) contains supplementary material, which is available to authorized users.

## Background

Vitamin D or cholecalciferol is a fat-soluble vitamin primarily synthesized from 7-dehydroxycholesterol in the skin by ultraviolet radiation [1]. A previous study showed that serum concentrations of 25-hydroxyvitamin D (25(OH)D) were related to geography and season [2]. Lower values were observed in the winter, whereas higher values were observed in the summer [3]. Additionally, a closer distance to the equator was associated with a lower magnitude of seasonal variation of 25(OH)D levels [4, 5]. This variation in vitamin D is resemblance to that in infection as the incidence and mortality of sepsis is highest during the winter months and is associated with increased respiratory illnesses [6]. The association of 25(OH)D deficiency and infections is likely due to the pleiotropic effects of 25(OH)D on human immunity, including T-cell proliferation, immunoglobulin class-switching, and cytokine release [7]. 1,25-dihydroxy vitamin D3 (1,25 (OH)_2_D) directly enhances signaling of the innate immune system to increase the production of antimicrobial peptides (AMP), such as cathelicidin and LL-37, its activated form [8]. Based on in vitro studies, LL-37 is active against various pathogens including Pseudomonas aeruginosa, Salmonella typhimurium, Escherichia coli, Listeria monocytogenes, Staphylococcus epidermidis, Staphylococcus aureus, and vancomycin-resistant enterococci [9]. The human cathelicidin (LL-37) has broad anti-microbial activities against bacteria, viruses and fungi.

A number of observational studies have found an association between low vitamin D levels and risk of sepsis. However, the effect of 25(OH)D in different infections is unclear and sepsis patients can have a heterogeneous mixture of pathogens and infected organ systems. The objective of this systematic review and meta-analysis of observational studies was to comprehensively determine the strength of association between vitamin D deficiency and sepsis in hospitalized patients, compared with optimal vitamin D group. The secondary objective was to determine any difference in 25(OH)D level between sepsis patients and controls.

## Methods

This systematic review and meta-analysis was conducted and reported according to established guidelines for meta-analysis of observational studies [10] and was registered in PROSPERO (registration number: CRD42014014767).

### Criteria for selection of studies for review

#### Types of studies

All published observational studies including prospective cohort, retrospective cohort, case-control, and cross-sectional studies, that evaluated association between levels of vitamin D (25(OH)D or 1,25-dihydroxyvitamin D [1,25(OH)_2_D]) or prevalence of vitamin D deficiency and the incidence or prevalence of sepsis during hospitalization were included. Reviews, case reports, letters, commentaries, abstracts, and unpublished studies were not included.

#### Types of participants

Participants of 18 years of age or older who were hospitalized and had vitamin D levels (25(OH)D or 1,25(OH)_2_D) measured before or during hospitalization were included.

#### Types of risk factors

We included all studies that defined vitamin D deficiency as a 25(OH)D level less than 20 ng/mL (50 nmol/L) [11] or defined an optimal level or vitamin D sufficiency as a 25(OH)D concentration greater than 30 ng/mL (75 nmol/L). The latter definition was used to characterize the optimal group. We reported definition of vitamin D deficiency and cut-off of 25(OH)D.

#### Types of outcome measures

Primary outcomes were incidence, prevalence, relative risk or odds ratio of having sepsis or bloodstream infection between patients with vitamin D deficiency and controls. We included studies that defined sepsis as a systemic inflammatory response syndrome caused by infection, or having International Classification of Diseases (ICD)-9 codes 038.0–038.9, 995.91, 995.92, or bloodstream infection. Studies that did not define sepsis were excluded. A difference in the levels of 25(OH)D between patients with sepsis and control was the secondary outcome.

#### Search methods for identification of studies

A.S. and S.U. independently searched published studies indexed in the Cochrane Central Register of Controlled Trials (CENTRAL) in The Cochrane Library, PubMed/MEDLINE, and EMBASE from database inception to October 2014. References of selected retrieved articles were also examined. Search terms that were used included: vitamin D, 25-hydroxyvitamin D, 25(OH)D, vitamin D2, vitamin D3, ergocalciferol, cholecalciferol, calcidiol, calcifediol, calcitriol, sepsis, septicemia, septic shock. Search terms that were used are detailed in Additional file 1.

### Data collection and analysis

#### Selection of studies

A.S. and S.U. independently reviewed the titles and abstracts of all citations identified. After all studies were abstracted, face-to-face data comparisons between investigators were conducted to ensure completeness and reliability. The inclusion criteria were independently applied to all identified studies. Only full-text articles in English were included in the meta-analysis. Differing decisions were resolved by consensus.

#### Data extraction and management

Full-text versions of potentially relevant papers identified in the initial screening were retrieved. Data concerning study design (cross-sectional, case-control, prospective cohort, retrospective cohort), participant characteristics (age, sex, settings), vitamin D levels, participants with vitamin D deficiency, and outcome measures (definition of sepsis, number of participants with sepsis, mortality rates, odds ratio or risk ratio) were independently extracted. We contacted authors of the primary reports to request any unpublished data. If the authors did not reply, we used the available data for our analyses.

#### Assessment of bias risk

The quality of observational studies was evaluated by two investigators (A.S. and S.U.) using the Newcastle-Ottawa quality assessment scale [12]. The NOS is based on three major components: selection of the study groups (0–4 stars in cohort study and 0-5 stars in cross-sectional study), comparability of cases and controls by controlling for relevant factors (0–2 stars), and ascertainment of exposure/outcome (0–3 stars). A study awarded 7 stars or more is considered a high-quality study, 4-6 stars is considered a moderate-quality study, and 0-3 stars is considered a poor-quality study. We excluded any studies that had poor quality from the meta-analysis.

#### Statistical methods

Data analysis was performed using Review Manager 5.3 software from The Cochrane Collaboration. We reported the pooled effect estimate (odds ratio) of sepsis comparing between vitamin D deficiency and optimal vitamin D groups. We also reported standardized mean difference of 25(OH)D levels between sepsis and control groups using a fixed effects model and inverse variance method. The heterogeneity of effect size estimates across these studies was quantified using the I^2^ statistic index and Cochran Q test [13]. Sensitivity analysis was conducted using random effects model. Publication bias was assessed visually using funnel plots of standard error vs odds ratio in the meta-analysis that included five or more studies [14].

## Results

### Description of included studies

**Fig. 1:**
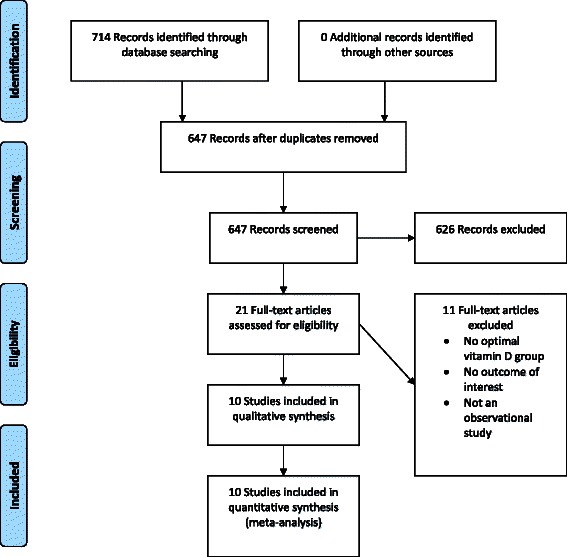
Results of the information search

The initial search yielded 647 articles (Fig. 1), of which 626 articles were excluded because they were not observational studies, participants did not have sepsis outcome, did not have vitamin D deficiency or vitamin D levels data, or were in a language other than English. A total of 21 articles underwent full-length review. Data were extracted from 10 observational studies [15–24] involving 33,810 participants. All extracted studies were included in the meta-analysis. The characteristics of the extracted studies are outlined in Table 1.

**Table 1:** Characteristics of included studies

Study	Design	Population characteristics	Age	Sepsis definition	Vitamin D cut-off (ng/mL)	Assay	Participants (n)	Time measure	25(OH)D (ng/mL)		1,25(OH)D2 (ng/mL)	
									Sepsis	Control	Sepsis	Control
Jeng 2009 [15]	Cross-sectional	Subjects in ICU with and without sepsis, and healthy controls between 1999 and 2006	54.0 (17.1)	ACCP and SCCM consensus panel in 2001	Insufficiency (<30)	ELISA	Total 70 Sufficiency 9 Insufficiency 61	During hospitalization	16.0 (8.5)	16.2 (7.2)	-	
Muller 2000 [16]	Cross-sectional	Patients admitted to MICU of the Basel University Hospitals	57 (15)	Systemic inflammatory response syndrome caused by infection	Subnormal (<20)	25(OH)D, Radioimmunoassay; 1,25(OH)D2, Scintillation proximity assay	Total 101	First 24 h, day 2, day of discharge	6.3 (5.2)	8.8 (7.7)	27.7 (23.5)	32.4 (15)
Su 2013 [17]	Cross-sectional	Patients admitted to ICU in Army Hospital in 2011	57 (20)	2001 International Sepsis Definition Conference	Deficiency (<20) Sufficiency (≥30)	High performance liquid chromatography and tandem mass spectrometry	Total 156	Within 24 h of ICU admission	0.91 (0.24)^a^	0.97 (0.22)^a^	-	
Braun 2012 [18]	Retrospective cohort	Patients aged ≥18 years who were admitted to BWH and MGH between 1997 and 2009	63.0 (17.2)	ICD-9-CM codes: 038.0–038.9, 790.7, 117.9, 112.5, 112.81, 995.92, and 785.52	Deficiency (≤15) Insufficiency (16–29) Sufficiency (≥30)	-	Total 1,325 Deficiency 668 Insufficiency 472 Sufficiency 185	7 days before and after critical care initiation	18.2 (13.7)		-	
Braun 2011[19]	Retrospective cohort	Patients aged ≥18 years who were admitted to BWH and MGH between 1997 and 2009	64.9 (16.6)	ICD-9 codes 038.0–038.9, 020.0, 790.7, 117.9, 112.5, 112.81	Deficiency (≤15) Insufficiency (16–29) Sufficiency (≥30)	-	Total 2,399 Deficiency 637 Insufficiency 918 Sufficiency 844	7-365 days before admission	26.4 (15.2)		-	
Flynn 2012 [20]	Prospective cohort study	Participants aged ≥18 years who were admitted to ICU ≥48 h for between 2010 -2011	56 (20)	-	Deficiency (<20) Sufficiency (≥20) Normal (30-100)	-	Total 66 Deficiency 49 Sufficiency 17	On admission and every 7 days during hospitalization	-		-	
Jovanovich 2014 [21]	Retrospective cohort	Hospitalized participants from hospitals and clinics from 2008-2010	60 (17)	ICD-9 codes 995.91, 995.92	Deficiency (<15) Insufficiency (15–30) Sufficiency (>30)	Radioimmunoassay	Total 132 Deficiency 74 Insufficiency 193 Sufficiency 252	3-15 months before admission	70.1 (62.2-79.6)	79.3 (71.1-88.1)	-	
Moromizato 2013 [22]	Retrospective cohort	Patients aged ≥18 years who were admitted to BWH and MGH between 1998 and 2011	65.9 (16.1)	Presence of any of the following ICD-9-CM codes: 038.0–038.9, 020.0, 790.7, 117.9, 112.5, or 112.81	Deficiency (≤15) Insufficiency (15–30) Sufficiency (≥30)	Chemiluminescence assay, radioimmunoassay, or mass spectroscopy	Total 3,386 Deficiency 566 Insufficiency 1,305 Sufficiency 1,515	7-365 days before admission	29.4 (15.5)		-	
Quraishi 2013 [23]	Retrospective cohort	Patients aged ≥18 years who were admitted to BWH and MGH between 1993 and 2011	61 (18)	Hospital-acquired bloodstream infection	Deficiency (<20)	Chemiluminescence assay, radioimmunoassay, or mass spectroscopy	Total 2,135	7-365 days before admission	25 (17)		-	
Lange 2013 [24]	Retrospective cohort	Patients aged ≥18 years who were admitted to BWH and MGH between 1993 and 2010	61.2 (17.6)	Community-acquired bloodstream infection	Deficiency (≤15) Insufficiency (15–30) Sufficiency (≥30)	Chemiluminescence assay, radioimmunoassay, or mass spectroscopy	Total 23,603 Deficiency 5,241 Insufficiency 8,679 Sufficiency 9,683	7-365 days before admission	27.9 (15.1)		-	

Data are presented as mean (S.D.) or median (interquartile range)

^a^Data in Su *et al.* are presented in logarithms of the 25-hydroxyvitamin D levels

ACCP, American College of Chest Physicians; BWH, Brigham and Women’s Hospital; MGH, Massachusetts General Hospital; SCCM, Society of Critical Care Medicine; MICU, medical intensive care unit; ICU, intensive care unit; 25(OH)D, 25-hydroxyvitamin D; 1,25(OH)D2, 1,25-dihydroxyvitamin D

Five studies defined vitamin D deficiency as 25(OH)D ≤15 ng/mL [18, 19, 21, 22, 24] and four studies defined as 25(OH)D ≤20 ng/mL [16, 17, 20, 23]. Only one study measured serum 1,25(OH)D2 and compared between sepsis and control groups [16]. In most studies, serum were drawn within 7-365 days before admission. Assays using for serum vitamin D measurement were ELISA, radioimmunoassay, scintillation proximity assay, tandem mass spectrometry, and chemiluminescence assay.

### Quality assessment

Quality assessment scores using the Newcastle-Ottawa Scale tool for observational studies are summarized in Table 2 and Table 3. Cross-sectional studies had a total score of 6-7 (selection scale of 2-3, comparability scale of 0-2, and exposure scale of 3). Cohort studies had a total score of 7-9 (selection scale of 4, comparability scale of 0-2, and exposure scale of 3).

**Table 2:** Quality assessment for cross-sectional study

Study (Year)	Selection						Comparability		Outcome				Total
	Representativeness of the sample		Justified sample size	Ascertainment of exposure		Comparable non-respondents rate between two groups	Comparability of different samples on the basis of the design or analysis		Assessment of outcome			Appropriate statistical test	
	truly representative	somewhat representative		Validated measurement tool	Non-validated tool		study controls for important factor	study controls for any additional factor	independent blind assessment		record linkage		
Jeng 2009				**			*	*	**			*	7
Muller 2000		*		**					**			*	6
Su 2013		*		**					**			*	6

**Table 3:** Quality assessment for cohort study

Study (Year)	Selection					Comparability		Outcome					Total
	Representativeness of the sample		Non-expose group from same community	Outcome of interest was not present at start of study	Validated measurement tool	Comparability of different samples on the basis of the design or analysis		Assessment of outcome		Follow up ≥1 month	Adequacy of follow up		
	truly representative	somewhat representative				study controls for important factor	study controls for any additional factor	independent blind assessment	record linkage		Complete	Small number loss follow-up	
Braun 2012	*		*	*	*	*	*		*	*		*	9
Braun 2011	*		*	*	*	*	*		*	*		*	9
Flynn 2012		*	*	*	*				*	*	*		7
Jovanovich 2014	*		*	*	*	*	*		*	*	*		9
Moromizato 2013	*		*	*	*	*	*		*	*		*	9
Quraishi 2013	*		*	*	*	*	*		*	*	*		9
Lange 2013	*		*	*	*	*	*		*	*	*		9

### Quantitative results (meta-analysis)

**Fig. 2:**
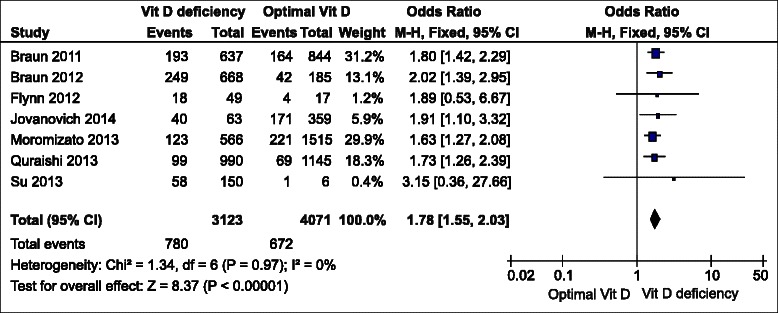
Forest plot of comparison of participants with sepsis between vitamin D deficiency (<20 ng/mL) and optimal groups (>30 ng/mL). CI, confidence interval; SE, standard error; Vit D, vitamin D

**Fig. 3:**
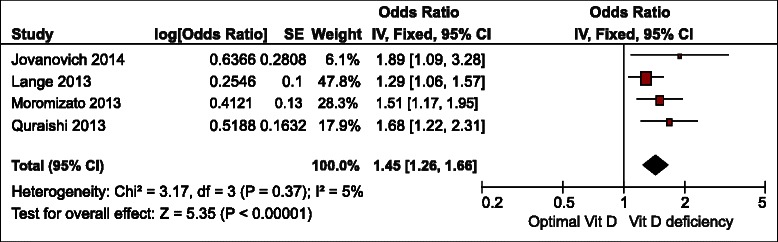
Forest plot of pooled odds ratio of included studies comparing vitamin D deficiency (<20 ng/mL) and optimal groups (>30 ng/mL). CI, confidence interval; Vit D, vitamin D

**Fig. 4:**
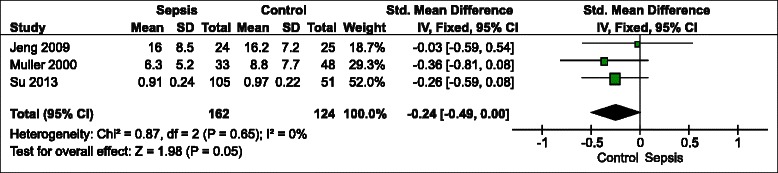
Forest plot of comparison of 25-hydroxyvitamin D (ng/mL) between sepsis and controls. CI, confidence interval; SD, standard deviation

The meta-analysis was performed using the fixed effects model (Fig. 2, 3, 4). It revealed that the pooled odds ratio of sepsis in participants with vitamin D deficiency compared with controls was 1.78 (95 % confidence interval [CI] = 1.55 to 2.03, p < 0.01) in studies that reported the number of participants with sepsis in vitamin D deficiency and control groups. In studies that reported an adjusted odds ratio of vitamin D deficiency for developing sepsis as an outcome, the pooled OR was 1.45 (95 % CI = 1.26 to 1.66, p < 0.01). The statistical between-study heterogeneity was low with an I^2^ of 0 % (Fig. 2) and 5 % (Fig. 3), respectively. Fig. 4 shows the standardized mean difference (SMD) of 25(OH)D levels in patients with sepsis and controls was −0.24 (95 % CI = −0.49 to 0.00, p = 0.05) and lower in the sepsis group compared with non-sepsis or control participants. The statistical between-study heterogeneity (I^2^) was 0 %.

### Sensitivity analysis

Sensitivity analysis was performed using the random effects model rather than the fixed effects model. The result of the direction of point estimate and statistical difference were not different from the main results.

### Meta-regression and subgroup analysis

Meta-regression and subgroup analysis were not performed because the between-study heterogeneity was minimal.

### Publication bias analysis

**Fig. 5:**
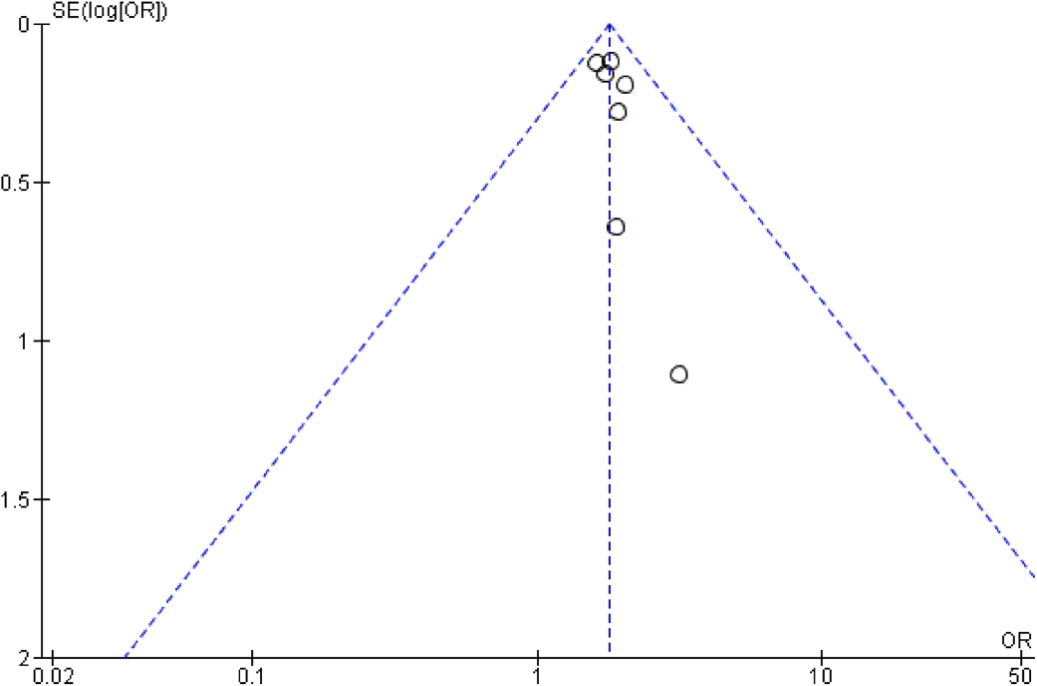
Funnel plots showing publication bias in the studies reporting number of participants with sepsis in vitamin D deficiency and optimal groups. Circles represent observed published studies

Potential publication bias was assessed by funnel plot of standard error vs odds ratio on the impact of vitamin D deficiency on sepsis (Fig. 5). It revealed a symmetrical distribution of included studies and did not show potential publication bias.

## Discussion

This systematic review and meta-analysis assessed the association between vitamin D deficiency, serum 25(OH) D level and sepsis. We observed that patients with vitamin D deficiency (25(OH)D < 15-20 ng/mL) measuring before or during hospitalization have higher odds of sepsis compared to individuals without vitamin D deficiency. We did not find a significant difference of 25(OH)D level between patients who had and did not have sepsis.

Low serum 25(OH)D levels are a common problem in the United States. Based on data from the US National Health and Nutrition Examination Survey (NHANES) in 2001–2006, two-thirds of the population had sufficient vitamin D. About one-quarter were at risk of vitamin D inadequacy, and 8 % were at risk of vitamin D deficiency [25]. The results of the current study reveal a statistically significant increased odds of vitamin D deficiency among septic patients. This meta-analysis finding is in agreement with other critical care studies’ findings. They revealed that low serum concentrations of 25(OH)D was associated with adverse clinical outcomes, including increased mortality rate, increased length of hospital stay, and acute kidney injury in a critical care setting not limited to severe infection and sepsis [26–28]. There were also evidences that deficiency in 25(OH)D and 1,25(OH)_2_D were significant predictors of 30-day mortality in septic patients [29, 30]. A pathophysiology that underlies cause of sepsis in vitamin D deficiency could be due to innate immune dysfunction [31]. Moreover, a reduction in 1,25(OH)_2_D at the tissue level may lower its pleiotropic effects on immune regulation, mucosal and endothelial function [32, 33].

Many randomized clinical studies did not find benefits on severe infections or sepsis after vitamin D normalization in vitamin D deficient patients [34–37]. The reason could be due to underpowered numbers, low dosages or short duration of supplementation. In most recent clinical trial of 475 patients with vitamin D deficiency [38], administration of high-dose vitamin D3 compared with placebo did not reduce hospital length of stay, hospital mortality, or 6-month mortality. However, hospital-mortality and mortality rates in this study were significantly lower in the severe vitamin D deficiency subgroup (25-hydroxyvitamin D level less than 12 ng/mL). Further clinical trial exploring effects of vitamin D supplementation on severe infection or sepsis in severe vitamin D deficiency patients is needed.

The results of this study should be cautiously interpreted because of several limitations in this meta-analysis of observational studies. A major limitation is the small number of studies that met our inclusion criteria. Additionally, analyzed results were from observational studies, which could contain potential confounders from factors in selected populations, such as age, gender, comorbidities, and prolonged hospitalization that might affect the risk of infection and having sepsis. Studies that measured serum 25(OH)D on admission or after hospitalization may not represent pre-illness vitamin D level. The vitamin D level could be underestimate from severe illness, or overestimate from vitamin D supplementation. We can assume that vitamin D level measuring before admission (at least 7 days) is not effected by critical illness. It is also a limitation to prevent sepsis by keeping optimal or normal vitamin D level in general population. It may also be difficult to normalize vitamin D in critically-ill patients.

## Conclusions

In conclusion, we confirm previous study findings that vitamin D deficiency by having serum 25(OH)D less than 15-20 ng/mL is associated with higher chance of sepsis. Larger prospective observational studies that measure vitamin D level before critical illness are required to determine the causal relationship of sepsis. Further randomized controlled trials are needed to determine the role of vitamin D normalization before or during critical illness, and to investigate whether it can prevent infection or improve clinical outcomes in infected organ systems.

## Additional file

Additional file 1: **Search methodology.** Details of the PubMed/MEDLINE, EMBASE, and CENTRAL database searches.
