# Supplementary material for: Significant association between vitamin D deficiency and sepsis: a systematic review and meta-analysis
Source: BMC Anesthesiol. 2015 Jun 4;15:84. doi: 10.1186/s12871-015-0063-3 (PMC4455341; doi:10.1186/s12871-015-0063-3)
Supplement: Additional file 1: — Search methodology. Details of the PubMed/MEDLINE, EMBASE, and CENTRAL database searches. [file 12871_2015_63_MOESM1_ESM.docx]

Additional file 1

Search Methodology

1. PubMed/MEDLINE

((((((Sepsis) OR septicemia) OR septic)) OR septicem*or septicaem*or seps*)) AND (((((vitamin d) OR ((((((((((("vitamin d"[MeSH Terms]) OR 25-hydroxyvitamin D) OR 1,25-dihydroxyvitamin D) OR 1,25(OH)(2)D) OR Vitamin D2) OR Vitamin D3) OR Ergocalciferol) OR Cholecalciferol) OR Calcidiol) OR calcifediol) OR Calcitriol)))))

1. EMBASE

(('vitamin d'/exp and [embase]/lim) or ('vitamin d' and [embase]/lim) or ('25 hydroxyvitamin d'/exp and [embase]/lim) or ('1 25 dihydroxyvitamin d'/exp and [embase]/lim) or ('vitamin d2'/exp and [embase]/lim) or ('vitamin d3'/exp and [embase]/lim) or ('25-hydroxyvitamin d' and [embase]/lim) or ('1,25-dihydroxyvitamin d' and [embase]/lim) or ('25(oh)d' and [embase]/lim) or ('1,25(oh)(2)d' and [embase]/lim) or ('vitamin d2' and [embase]/lim) or ('vitamin d3' and [embase]/lim) or ('ergocalciferol' and [embase]/lim) or ('cholecalciferol' and [embase]/lim) or ('calcitriol' and [embase]/lim) or ('calcidiol' and [embase]/lim) or ('calcifediol' and [embase]/lim)) and (('sepsis'/exp or sepsis and [embase]/lim) or ('septic shock'/exp or 'septic shock' and [embase]/lim) or ('septicemia'/exp or 'septicemia' and [embase]/lim) or (septicaemia and [embase]/lim))

1. CENTRAL

#1 MeSH descriptor: [Vitamin D] explode all trees

#2 vitamin d

#3 25-hydroxyvitamin D

#4 1,25-dihydroxyvitamin D

#5 1,25 (OH) (2) D

#6 25 (OH) D

#7 Vitamin D2

#8 Vitamin D3

#9 Ergocalciferol

#10 Cholecalciferol

#11 Calcidiol

#12 calcifediol

#13 Calcitriol

#14 #1 or #2 or #3 or #4 or #5 or #6 or #7 or #8 or #9 or #10 or #11 or #12 or #13

#15 MeSH descriptor: [Sepsis] explode all trees

#16 MeSH descriptor: [Shock, Septic] explode all trees

#17 MeSH descriptor: [Sepsis] explode all trees

#18 septicem*

#19 septicaem*

#20 seps*

#21 sept* near/6 shock*

#22 #15 or #16 or #17 or #18 or #19 or #20 or #21

#23 #14 and #22
